# Regulatory Action of Calcium Ion on Cyclic AMP-Enhanced Expression of Implantation-Related Factors in Human Endometrial Cells

**DOI:** 10.1371/journal.pone.0132017

**Published:** 2015-07-10

**Authors:** Kazuya Kusama, Mikihiro Yoshie, Kazuhiro Tamura, Kazuhiko Imakawa, Keiichi Isaka, Eiichi Tachikawa

**Affiliations:** 1 Department of Endocrine and Neural Pharmacology, Tokyo University of Pharmacy and Life Sciences, 1432–1 Horinouchi, Hachioji, Tokyo, 192–0392, Japan; 2 Laboratory of Theriogenology and Animal Breeding, Graduate School of Agricultural and Life Sciences, The University of Tokyo, 1-1-1 Yayoi, Bunkyo, Tokyo, 113–8657, Japan; 3 Department of Obstetrics and Gynecology, Tokyo Medical University, 6-7-1 Nishishinjuku, Tokyo, 160–0023, Japan; Indiana University School of Medicine, UNITED STATES

## Abstract

Decidualization of human endometrial stroma and gland development is mediated through cyclic AMP (cAMP), but the role of intracellular calcium ion (Ca^2+^) on cAMP mediated-signaling in human endometrial stroma and glandular epithelia has not been well-characterized. The present study was designed to investigate the role of intracellular Ca^2+^ on cAMP mediated-decidualization and gland maturation events, which can be identified by the up-regulation of prolactin and IGF-binding protein (IGFBP)1 in human endometrial stromal cells (ESCs), and cyclooxygenase 2 (COX2) and prostaglandin E2 (PGE2) and glandular epithelial EM-1 cells. Increases in decidual *prolactin* and *IGFBP-1* transcript levels, induced by cAMP-elevating agents forskolin or dibutyryl cyclic AMP, were inhibited by Ca^2+^ influx into ESCs with Ca^2+^ ionophores (alamethicin, ionomycin) in a dose-dependent manner. Conversely, inhibitors of Ca^2+^ influx through L-type voltage-dependent Ca^2+^ channel (VDCC), nifedipine and verapamil, enhanced the decidual gene expression. Furthermore, dantrolene, an inhibitor of Ca^2+^ release from the intracellular Ca^2+^ store, up-regulated *prolactin* and *IGFBP-1* expression. Ca^2+^ ionophores decreased intracellular cAMP concentrations, whereas nifedipine, verapamil or dantrolene increased cAMP concentrations in ESCs. In glandular epithelial cells, similar responses in *COX2* expression and PGE2 production were found when intracellular cAMP levels were up-regulated by decreases in Ca^2+^ concentrations. Thus, a marked decrease in cytosolic Ca^2+^ levels caused the elevation of cAMP concentrations, resulting in enhanced expression of implantation-related factors including decidual markers. These findings suggest that fluctuation in cytosolic Ca^2+^ concentrations alters intracellular cAMP levels, which then regulate differentiation of endometrial stromal and glandular epithelial cells.

## Introduction

Receptive endometrium for implantation is constituted with the luminal epithelium, decidual cells, and glandular epithelial cells which secrete substances that support blastocyst development. Uterine endometrial stromal cells (ESCs) differentiate into decidual cells, called as decidualization during the secretory phase of the menstrual cycle. Decidualization of ESCs occurs spontaneously during the menstrual cycles. This differentiation is indispensable for successful embryo implantation and subsequent placenta formation [1]. One of the hallmarks of decidualization induction is the expression of specific marker gene expression such as prolactin [2] and IGF-binding protein (IGFBP) 1 [3]. Decidual cells and large glandular lymphocytes modulate trophoblast function and endometrial preparation including angiogenesis through the secretion of various cytokines and growth factor-binding protein. The endometrial glands are tortuous in the mid-secretory and late secretory phases. Their secretory activity reaches a maximum after ovulation, and the structural transformation and differentiation of the glandular epithelium occur in the functionalis layer of the endometrium during early pregnancy in human [4]. Decidualization of ESCs is mainly induced by ovarian steroids [5, 6], and progesterone-dependent decidualization is mediated in part by the second messenger cAMP [7, 8]. This process is enhanced by physiological factors modulating adenylyl cyclase (AC) activity through receptors functionally coupled with Gs proteins such as prostaglandin (PG) E2 [9] and relaxin [10], or by a cAMP analog [5]. cAMP triggers intracellular signaling pathways that affect diverse downstream molecules. It has been documented that decidualization is mainly regulated by both protein kinase A (PKA) and exchange protein directly activated by cAMP (EPAC) signalings [11–13]. These data reveal that cAMP is a key mediator of decidualization in ESCs. In addition, endometrial glandular epithelial cells synthesize and secrete implantation-related factors including PGE2 during the implantation window, which are essential for embryo development and endometrial stromal cell differentiation [14, 15]. Activation of the cAMP signaling increases cyclooxygenase (COX) 2 expression in endometrial glandular cells [16]. It has been demonstrated that both cAMP/PKA and cAMP/EPAC signaling control the function of endometrial glandular cells [17].

Similar to the cAMP signaling, intracellular calcium ions (Ca^2+^) have been shown to play an essential role as a second messenger in various physiological and pharmacological systems. Calcium-mobilizing mechanism exists in the cells, including Ca^2+^ influx from the extracellular region and Ca^2+^ release into cytoplasm from internal stores such as endoplasmic reticulum (ER) [18]. Vital roles of Ca^2+^ homeostasis in endometrial differentiation and implantation have been reported in human ESCs [19, 20]. The transient receptor potential canonical (TRPC) channel, a member of the non-voltage-dependent Ca^2+^ channel (non-VDCC) superfamily, induces *IGFBP1* expression via Ca^2+^ influx [19]. In uterine epithelial cells, S100A11, a Ca^2+^-binding protein, is involved in the process of embryo implantation [20]. Furthermore, the activation of the epithelial Na^+^ channel triggers Ca^2+^ influx, and leads to the up-regulation of *COX2* expression and PGE2 release via the activation of PKA in mouse uterine epithelial cells [21]. These findings indicate that intracellular Ca^2+^ signal could be closely associated with the preparation of endometrium for embryo implantation. Despite the importance of Ca^2+^ and cAMP on endometrial differentiation, the relationship between Ca^2+^ and cAMP in the endometrium has not been studied. This study investigated whether Ca^2+^ plays a role on endometrial differentiation mediated by cAMP signaling in human stromal and glandular epithelial cells.

## Materials and Methods

### Reagents

A cAMP analog N^6^, 2′-O-dibutyryladenosine 3′, 5′-cyclic monophosphate (db-cAMP) and various Ca^2+^ modulators nifedipine, verapamil, dantrolene, alamethicin, and ionomycin were purchased from Sigma-Aldrich (St. Louis, MO). Forskolin, an activator of AC, was obtained from Applichem (Darmstadt, Germany). *O*,*O'*-Bis (2-aminophenyl)ethyleneglycol-*N*,*N*,*N'*,*N'*-tetraacetic acid, tetraacetoxymethyl ester; BAPTA)was commercially provided by Dojindo (Kumamoto, Japan)

### Isolation of human endometrial stromal cells (ESCs), the culture of ESCs and glandular epithelial cell line (EM-1), and reagent treatments

Samples of eutopic endometrial tissue in the proliferative phase (n = 6) were obtained from women undergoing endometriosis surgery. The patient signed an informed consent and accepted to participate to this research project, which was approved by the clinical research ethics committee of the Tokyo Medical University Hospital and the Tokyo University of Pharmacy and Life Sciences. Primary ESCs were obtained as described previously [12]. To elucidate a potential role of Ca^2+^ on the production of cAMP signaling-induced factor in human endometrium, ESCs and immortalized human endometrial glandular epithelial cell line (EM-E6/E7/TERT-1 cells; EM1) [22] were subjected to this study. ESCs or EM1 cells plated at a density of 2 x 10^4^ cells/cm^2^ were grown at 37°C in Dulbecco’s modified Eagle medium and Ham’s F-12 supplemented with 10% (w/v) charcoal-stripped fetal bovine serum, 50 μg/ml penicillin, 50 μg/ml streptomycin, 100 μg/ml neomycin, and 0.5 μg/ml amphotericin B [12, 17]. In preliminary experiments to validate the experimental condition, the expression of decidual markers, *IGFBP1* and *prolactin* expression peaked within 5 days (120 h) with progression of decidualization in ESC [23, 24]. Epithelial *COX2* expression and PGE2 accumulation into the media increased at 6 h after PKA activator and reached to the maximum levels between 48 and 72 h in a preliminary experiment. ESCs or EM1 cells were, therefore, treated with the above Ca^2+^ modulators for 1 h and then stimulated with forskolin or db-cAMP for 48 h.

### RNA extraction and real-time RT-PCR

Total RNA was extracted from endometrial cells using Isogen reagent (Nippon Gene, Tokyo Japan) and quantified by A260/A280 measurement using a NanoVue (GE Healthcare Bioscience, Tokyo). Real time PCR was performed with the iQ5 Real time PCR Detection System (Bio-Rad) in triplicate in 20 μl volumes containing 100 ng RNA, 10 μl 2 X SYBR Green Master Mix (iScript One-Step RT-PCR Kit; Bio-Rad Laboratories, Hercules, CA) and 50 nM of primers. Human glyceraldehyde 3-phosphate dehydrogenase (GAPDH) mRNA was used as an internal standard for RNA loading. The following specific sense (S) and antisense (AS) primers were used: *Prolactin*; 5’-AAAGGATCGCCATGGAAAG-3’ (S) and 5’-GGTCTCGAAGGGTCACCTG-3’ (AS), *IGFBP1*; 5’-AATGGATTTTATCACAGCAGACAG-3’ (S) and 5’-GGTAGACGCACCAGCAGAGT-3’ (AS), *COX*2; 5’-CTTCACGCATCAGTTTTTCAAG-3’ (S) and 5’-TCACCGTAAATATGATTTAAGTCCAC-3’ (AS), and *GAPDH*; 5’-AGCCACATCGCTCAGACA-3’ (S) and 5’-GCCCAATACGACCAAATCC-3’ (AS). The PCR consisted 40 cycles at 95 C for 10 s, annealing and extension at 60 C at 30s. iQ5 Optical system software was used to collect the data and calculate the threshold cycle (Ct). The expression of each mRNA was normalized with GAPDH and analyzed by the comparative Ct method [17].

### PGE2 and IGFBP1 ELISA

EM1 cells were treated with alamethicin, ionomycin, nifedipine, verapamil, or dantrolene for 1 h and then with forskolin or db-cAMP for 48 h. The culture medium (500 μl) was centrifuged at 10,000 x g at 4°C for 10 min, and the concentration of PGE2 in the supernatant was determined using a ELISA kit (Prostaglandin E2 Express EIA kit, Cayman Chemical Company, Ann Arbor, MI). One hundred μl of the supernatant was diluted 2-fold with EIA buffer for each sample measurement. Three independent sets of experiments were performed in triplicate. For IGFBP measurement at protein levels, the culture medium was centrifuged as above and IGFBP-1 in the supernatant was determined using a commercially available sandwich ELISA kit (human IGFBP-1 DuoSet kit, R&B Systems, Inc., Minneapolis, MN), as described before [11]. The concentration of IGFBP-1 was normalized to the amount of total cell protein.

### cAMP assay

Total cAMP levels in ESCs and EM1 cells, treated with forskolin in the presence of various Ca^2+^ modulators for 48 h, were determined using a competitive EIA kit (Cyclic AMP EIA kit, Cayman Chemical Company) according to the manufacturer’s recommendations. Briefly, cells were lysed for 10 min in 80 μl of 0.1 M HCl and were centrifuged at 1,000 x g at 4°C. Sixty μl of supernatant was used for the measurement. The values for inter- and intra-assay coefficients for EIA were below 10%.

### Cell viability assay

Cells plated at a density of 6 x 10^3^ cells/cm^2^ in 96 well dish were treated with vehicle or various inhibitors for 48 h in the presence or absence of forskolin (15 μM). After treatment, cell viability was assessed using the WST-8 (the tetrazolium reagent 2-(2-methoxy-4-nitrophenyl)-3-(4-nitrophenyl)-5-(2, 4-disulfophenyl)-2H-tetrazolium; Cell counting kit, Dojindo, Kumamoto, Tokyo). Culture medium was removed and 100 μl of WST-8 (1:10 dilution) in phosphate buffered saline were added to each well following each inhibitor treatment. After incubation in CO_2_ incubator for 1 h, 50 μl from each well were then transferred to a 96-well microplate and read at 450 nm.

### Statistical analysis

Data are expressed as the mean ± SEM. Significance was assessed using the Tukey-Kramer multiple comparisons test. A *P*-value < 0.05 was considered statistically significant.

## Results

### Ca^2+^ influx inhibited cAMP elevating agents-induced decidualization in ESCs

**Fig 1:**
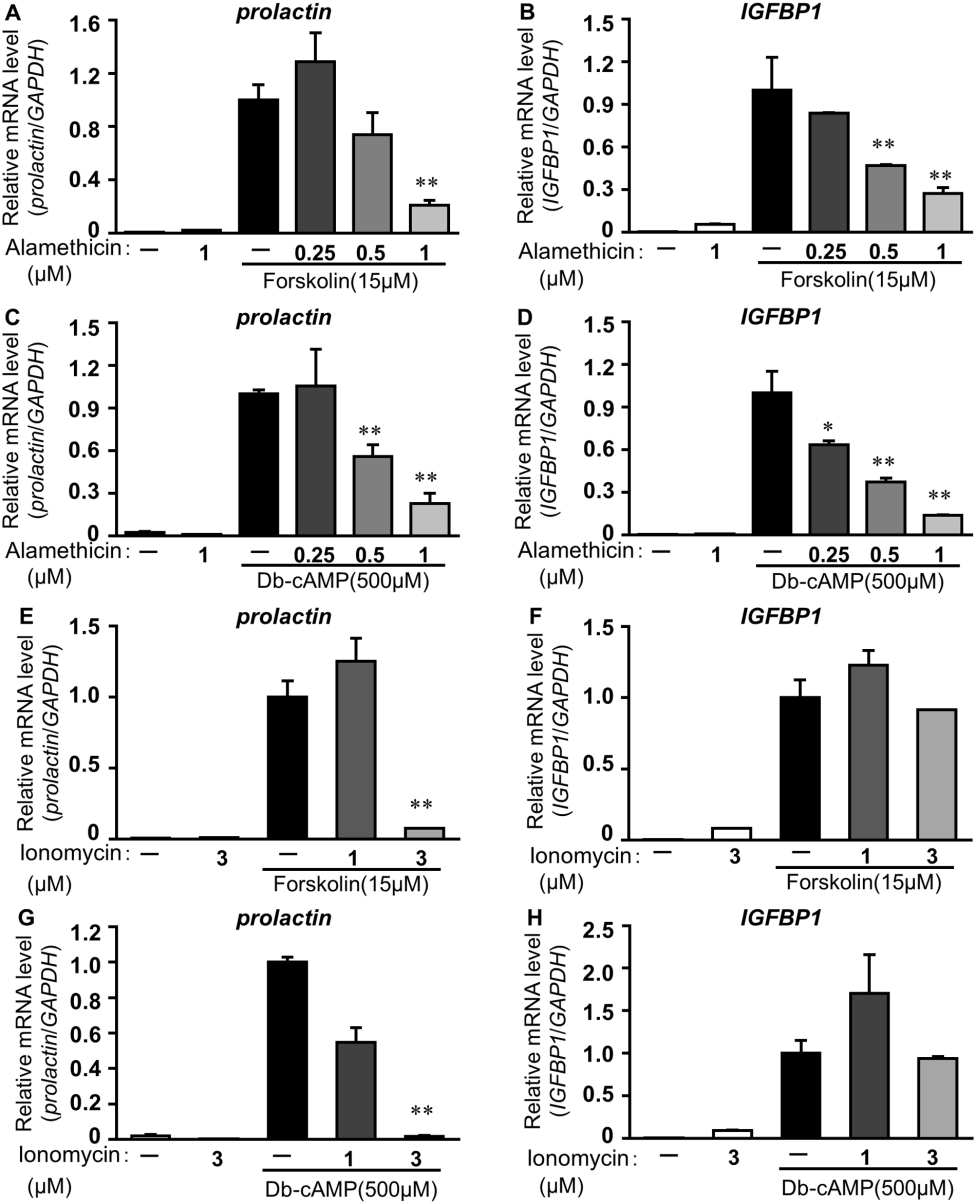
Ca^2+^ influx inhibits the decidual markers expression in ESCs. ESCs were treated for 1 h with alamethicin (A-D: 0.25, 0.5, 1 μM) or ionomycin (E-H: 1, 3 μM) and then cultured for 48 h with forskolin (A, B, E, F: 15 μM) or db-cAMP (500 μM). Total RNA was subjected to real-time RT-PCR analysis to determine *prolactin* and *IGFBP1* mRNA levels. *GAPDH* was used as an internal control. The data from three independent experiments are presented. **p<0.01, *p<0.05 *vs*. forskolin or db-cAMP alone. Values represent the mean ± SEM.

To investigate whether Ca^2+^ influx into the cytoplasm affected decidual marker expression, ESCs were pretreated with alamethicin or ionomycin that transports Ca^2+^ across the lipid bilayer of cell membrane, and were then stimulated with a general activator of AC, forskolin or db-cAMP. Forskolin enhanced *prolactin* (161 ± 18 fold) and *IGFBP1* (1927 ± 443 fold) mRNA levels, compared with control levels. Pretreatment with alamethicin reduced forskolin-induced *prolactin* (Fig 1A) or *IGFBP1* (Fig 1B) expression in a concentration-dependent manner. Significant decreases in *prolactin* and *IGFBP1* were obtained at 1 μM alamethcin and 0.5 or 1 μM alamethicin, respectively. (Fig 1A and 1B). Alamethicin also blocked db-cAMP-stimulated *prolactin* and *IGFBP1* expression (Fig 1C and 1D). Similar to alamethicin, ionomycin inhibited *prolactin* (0.07 ± 0.002), but not *IGFBP1* expression induced by forskolin (0.92 ± 0.001) (Fig 1E and 1F). Pretreatment with ionomycin also reduced db-cAMP-stimulated *prolactin* expression (0.02 ± 0.005) (Fig 1G). No effect on db-cAMP-stimulated *IGFBP1* was observed in the ionomycin-treated group (Fig 1H).

### L-type voltage-dependent Ca^2+^ channel (VDCC) blockers promoted the decidual marker expression in ESCs

**Fig 2:**
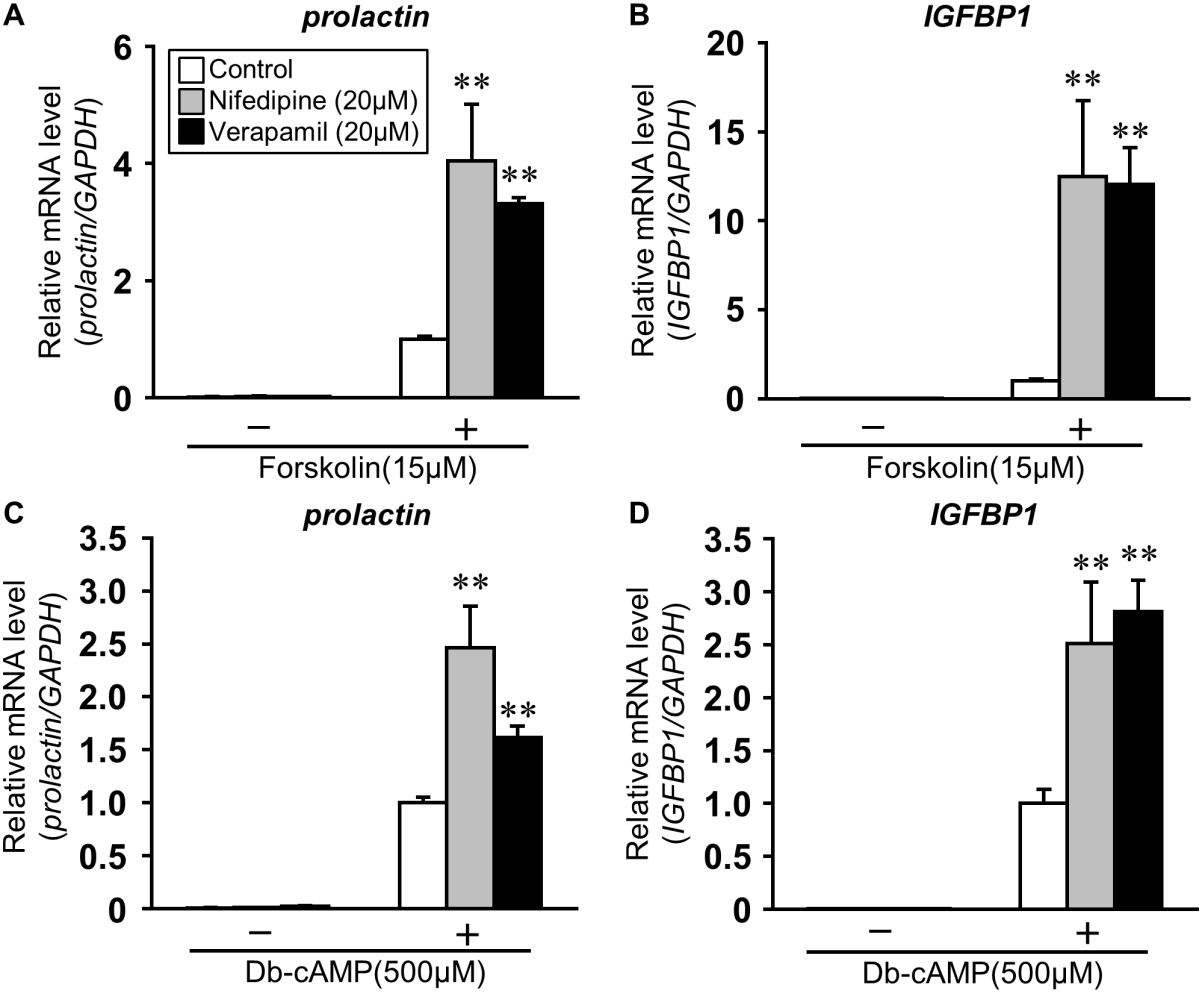
VDCC blockers promote the decidual markers expression in ESCs. ESCs were treated for 1 h with nifedipine (20 μM) or verapamil (20 μM) and then stimulated for 48 h with forskolin (A, B: 15 μM) or db-cAMP (C, D: 500 μM). Total RNA was subjected to real-time RT-PCR analysis to determine *prolactin* and *IGFBP1* mRNA levels. *GAPDH* was used as an internal control. The data from three independent experiments are presented. **p<0.01 *vs*. forskolin or db-cAMP alone. Values represent the mean ± SEM.

To further study the effects of continuous Ca^2+^ influx into the cytoplasm from extracellular region, the effect of VDCC blockers of dihydropyridines (DHP) type such as nifedipine or non-DHP of verapamil on the AC/cAMP pathway-mediated decidual marker expression was examined. Nifedipine or verapamil pretreatment promoted forskolin-induced expression of *prolactin* in ESCs (4.04 ± 0.97 in nifedipine, 3.31 ± 0.11 in verapamil *vs*. control levels; p<0.01, Fig 2A). Both blockers also enhanced *IGFBP1* expression (12.48 ± 4.27 in nifedipine, 12.04 ± 2.01 in verapamil *vs*. control; p<0.01, Fig 2B). Furthermore, both inhibitors significantly enhanced db-cAMP-induced expression of *prolactin* (2.46 ± 0.40 in nifedipine, 1.61 ± 0.11 in verapamil *vs*. control, Fig 2C) and *IGFBP1* (2.51 ± 0.58 in nifedipine, 2.81 ± 0.30 in verapamil *vs*. control, Fig 2D).

### Ryanodine receptor inhibitor, dantrolene stimulated the expression of decidual markers in ESCs

**Fig 3:**
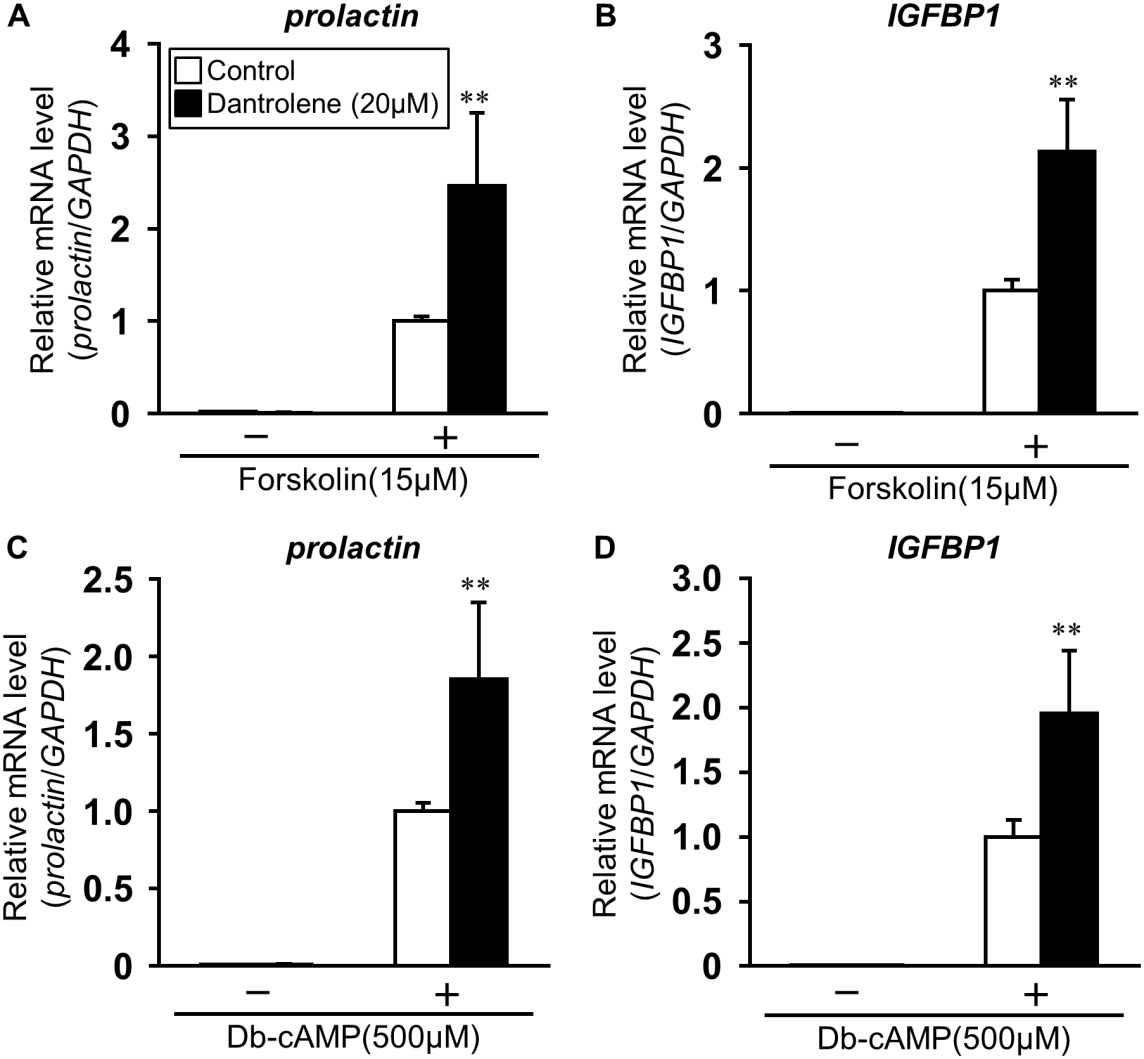
Ryanodine receptor (RyR) inhibitor enhances decidual markers expression in ESCs. ESCs were treated for 1 h with dantrolene (20 μM) and then stimulated for 48 h with forskolin (A, B: 15 μM) or db-cAMP (C, D: 500 μM). Total RNA was subjected to real-time RT-PCR analysis to determine *prolactin* and *IGFBP1* mRNA levels. *GAPDH* was used as an internal control. The data from three independent experiments are presented. **p<0.01 *vs*. forskolin or db-cAMP alone. Values represent the mean ± SEM.

Because the inhibition of VDCC promoted the stimulatory effect of cAMP on decidualization, we further examined the effect of dantrolene, a blocker of the ryanodine receptor (RyR) that inhibits the release of Ca^2+^ from ER into cytoplasm, on the expression of *prolactin* (Fig 3A and 3C) and *IGFBP1* (Fig 3B and 3D) in ESCs. Pretreatment with dantrolene significantly promoted the expression of both *prolactin* (2.46 ± 0.79) and *IGFBP1* (2.13 ± 0.42) induced by forskolin (Fig 3A and 3B). Similar to the condition of forskolin stimulation, dantrolene further enhanced *prolactin* (1.85 ± 0.50) and *IGFBP1* (1.95 ± 0.49) expression in the presence of db-cAMP (Fig 3C and 3D). Inhibitors of Ca^2+^ influx into cytoplasm (nifedipine, verapamil, and dantrolene, 20 μM) as well as the ionophore, alamethicin (1 μM) and ionomycin (3 μM) did not affect cell viability, although ionomycin at 10 μM was found to be cytotoxic to ESCs (S1 Fig). Furthermore, the levels of IGFBP1 in media were significantly increased by forskolin stimulation for 48 h, compared with control (forskolin: 2.55 ± 0.10 ng/ml vs. control: 0.071 ± 0.08 ng/ml) (S2 Fig). Pretreatment with nifedipine, verapamil, or dantrolene promoted forskolin-induced expression of *IGFBP1*, whereas alamethicin inhibited the stimulatory effect of forskolin in ESCs.

### VDCC blockers and dantrolene altered the concentration of cAMP in ESCs

**Fig 4:**
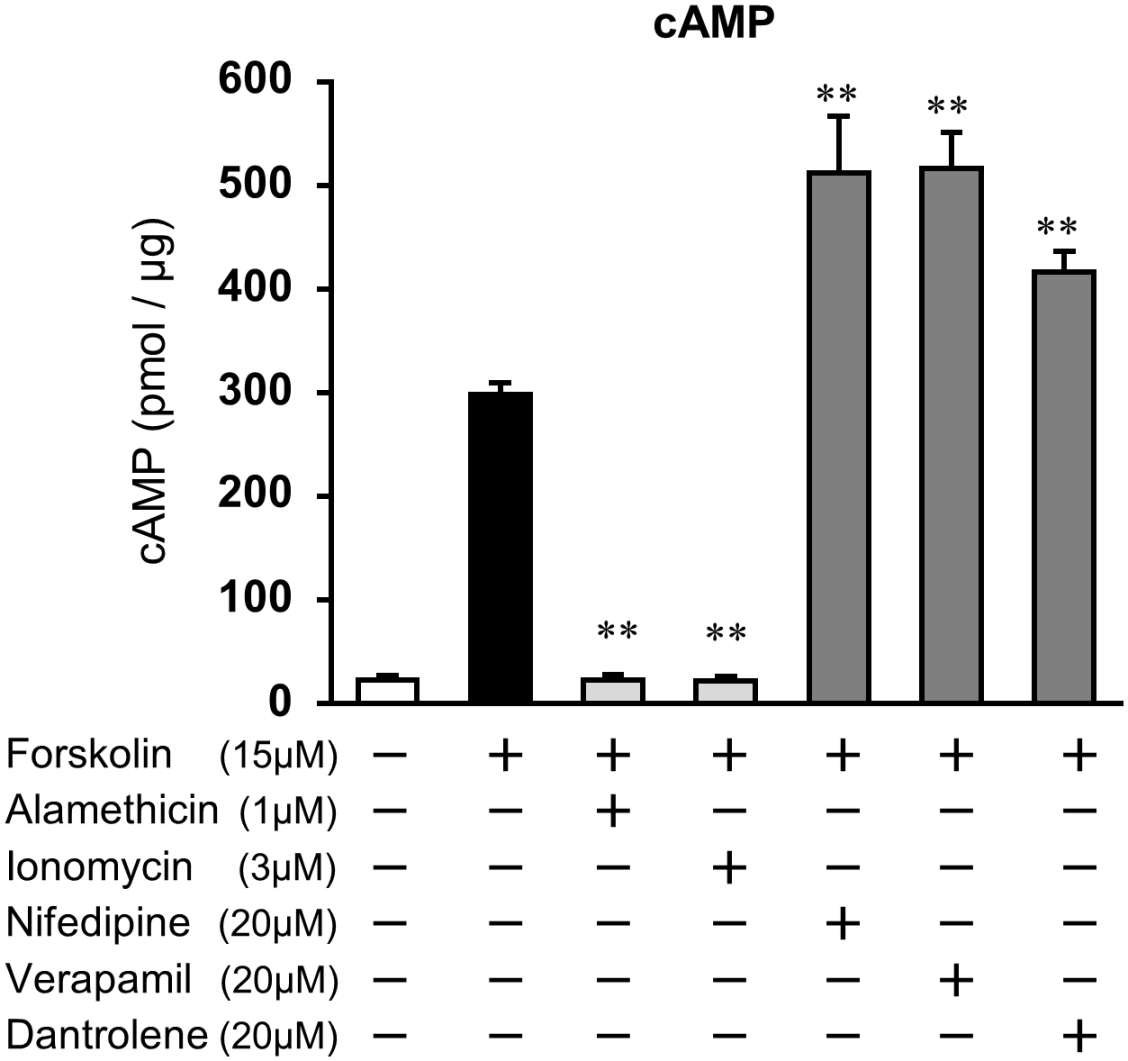
Ca^2+^ modulators alter the concentrations of cAMP in ESCs. ESCs were treated for 1 h with alamethicin (1 μM), ionomycin (3 μM), nifedipine (20 μM), verapamil (20 μM), or dantrolene (20 μM) and then stimulated for 48 h with forskolin (15 μM). The cAMP levels in the cell lysates were determined by EIA. The amount of cAMP was normalized to the amount of total cellular protein. The data from three independent experiments are presented. **p<0.01 *vs*. forskolin alone-treated group. Values represent the mean ± SEM.

We hypothesized that increases in cytosolic Ca^2+^ lower the levels of cAMP, resulting in the down-regulation of *prolactin* and *IGFBP1* expression in ESCs. To test this hypothesis, the effect of the inhibitors or accelerators of intracellular Ca^2+^ influx on cAMP concentration was examined in ESCs (Fig 4). Alamethicin or ionomycin pretreatment significantly decreased forskolin-induced elevation of cAMP accumulation (22.5 ± 5.2 pmol/μg in alamethicin, 21.9 ± 4.8 pmol/μg in ionomycin; p<0.01 *vs*. forskolin, 300 ± 4.8 pmol/μg). The forskolin-stimulated cAMP levels were significantly enhanced by nifedipine (511.9 ± 55.1 pmol/μg), verapamil (516.6 ± 34.4 pmol/μg), or dantrolene (416.6 ± 20.2 pmol/μg), compared with those in the forskolin-treated group.

### VDCC blockers and dantrolene influenced the forskolin-induced PGE2 production in EM1 cells

**Fig 5:**
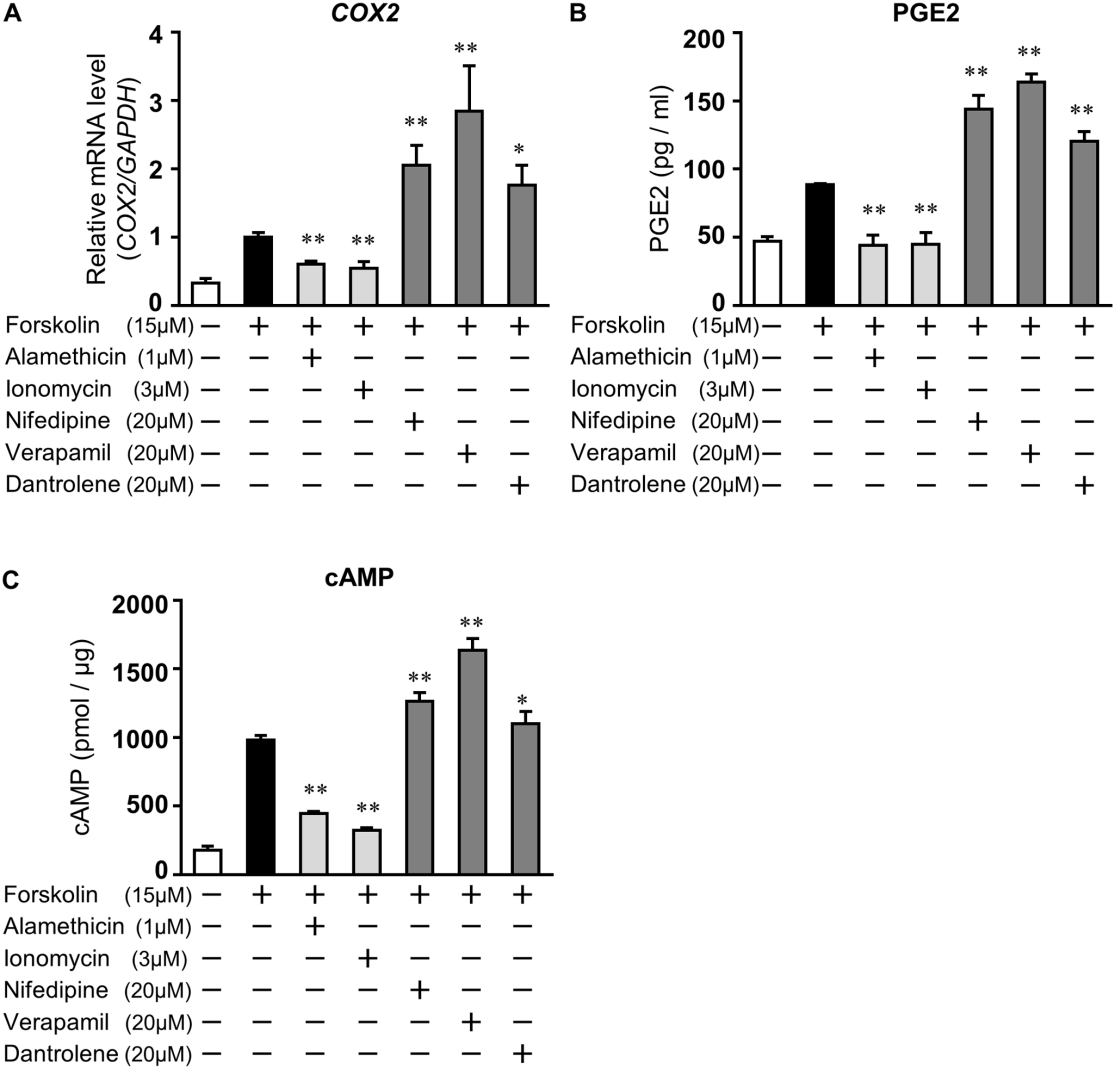
Ca^2+^ modulators affect the cAMP-induced *COX2* expression and PGE2 production in epithelial EM1 cells. EM1 cells were treated for 1 h with alamethicin (1 μM), ionomycin (3 μM), nifedipine (20 μM), verapamil (20 μM), or dantrolene (20 μM) and then stimulated for 48 h with forskolin (15 μM). A. Total RNA was subjected to real-time RT-PCR analysis to determine *COX2* mRNA levels. *GAPDH* was used as an internal control. The data from three independent experiments are presented. **p<0.01, *p<0.05 *vs*. forskolin alone. B. The PGE2 levels released into media were determined by EIA. The data from three independent experiments are presented. **p<0.01 *vs*. forskolin alone. Values represent the mean ± SEM. C. The cAMP levels in the cell lysates were determined by EIA. The data from three independent experiments are presented. **p<0.01, *p<0.05 *vs*. forskolin alone. Values represent the mean ± SEM.

We and others have shown that the activation of cAMP signaling stimulates the expression of *COX2* and then PGE2 production in endometrial glandular epithelial cells [16, 17]. To investigate whether the alteration of cytosolic Ca^2+^ concentration affected cAMP signaling-induced *COX2* expression and PGE2 secretion in EM1 cells, the effect of Ca^2+^ modulators on *COX2* expression and PGE2 secretion was examined. Forskolin stimulated *COX2* expression (3.09 ± 0.07; p<0.05 vs. no treatment, Fig 5A) and PGE2 secretion (88.7 ± 0.6 pg/ml; p<0.05 *vs*. no treatment, Fig 5B). However, pretreatment with alamethicin and ionomycin inhibited forskolin-induced *COX2* expression (0.60 ± 0.05 and 0.67 ± 0.04, respectively) (Fig 5A) and PGE2 secretion (43.2 ± 6.5 pg/ml and 44.8 ± 8.0 pg/ml, respectively: p<0.05 *vs*. forskolin) (Fig 5B). However, pretreatment with VDCC blockers or dantrolene promoted forskolin-induced *COX2* expression (alamethicin: 2.05 ± 0.29, ionomycin: 2.84 ± 0.67, and dantrolene: 1.76 ± 0.29; p<0.05) and PGE2 secretion (143.9 ± 10.2 pg/ml, 163.8 ± 5.8 pg/ml and 120.4 ± 7.2 pg/ml; p<0.05). Furthermore, as shown in Fig 5C, forskolin-induced cAMP elevation was significantly reduced by the treatment with alamethicin (446.8 ± 12.6 pmol/μg) or ionomycin (321.8 ± 19.6 pmol/μg), but enhanced by treatment with nifedipine (1262.3 ± 65.7 pmol/μg), verapamil (1635.9 ± 86.6 pmol/μg) or dantrolene (1100.7 ± 87.1 pmol/μg).

### Ca^2+^ chelator counteracted alamethicin-induced inhibition of decidual marker and COX2 in ESCs and EM1 cells

**Fig 6:**
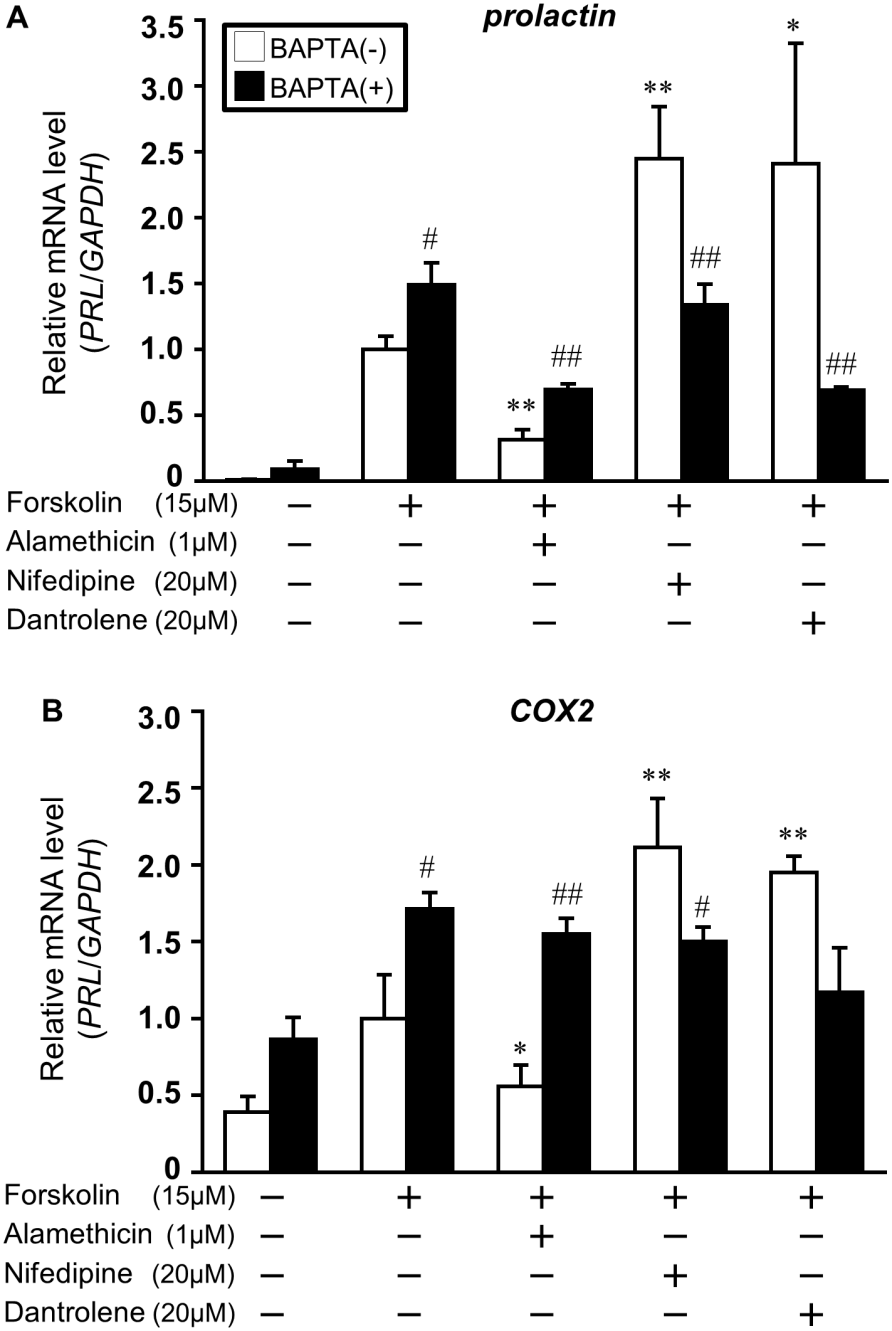
Ca^2+^ chelator counteracted the effect of Ca^2+^ ionophore in ESCs and EM1 cells. ESCs or EM1 cells were treated for 1 h with alamethicin (1 μM), nifedipine (20 μM), or dantrolene (20 μM) a in the presence of BAPTA (20 μM) and then stimulated with forskolin (15 μM). Total RNA was subjected to real-time RT-PCR analysis to determine *prolactin* mRNA levels in ESCs (A) or *COX2* mRNA levels in EM1 cells (B). *GAPDH* was used as an internal control. The data from three independent experiments are presented. **p<0.01, *p<0.05 *vs*. forskolin alone. ^##^p<0.01, ^#^p<0.05 *vs*. treatment without BAPTA (BAPTA(-)). Values represent the mean ± SEM.

To explore the role of intracellular Ca^2+^ in endometrial differentiation, we examined whether Ca^2+^ chelator, BAPTA influences the efficiency of alamethicin, nifedipine, or dantrolene on forskolin-induced *prolactin* and *COX2* expression in ESCs and EM1 cell. BAPTA counteracted the down-regulation of forskolin-induced *prolactin* and *COX2* by alamethicin (Fig 6A and 6B). Interestingly, BAPTA increased forskolin-induced *prolactin* (1.49 ± 0.17) and *COX2* (1.71 ± 0.11) expression, whereas the expression of *prolactin* and *COX2* induced by nifedipine or dantrolene was suppressed.

## Discussion

**Fig 7:**
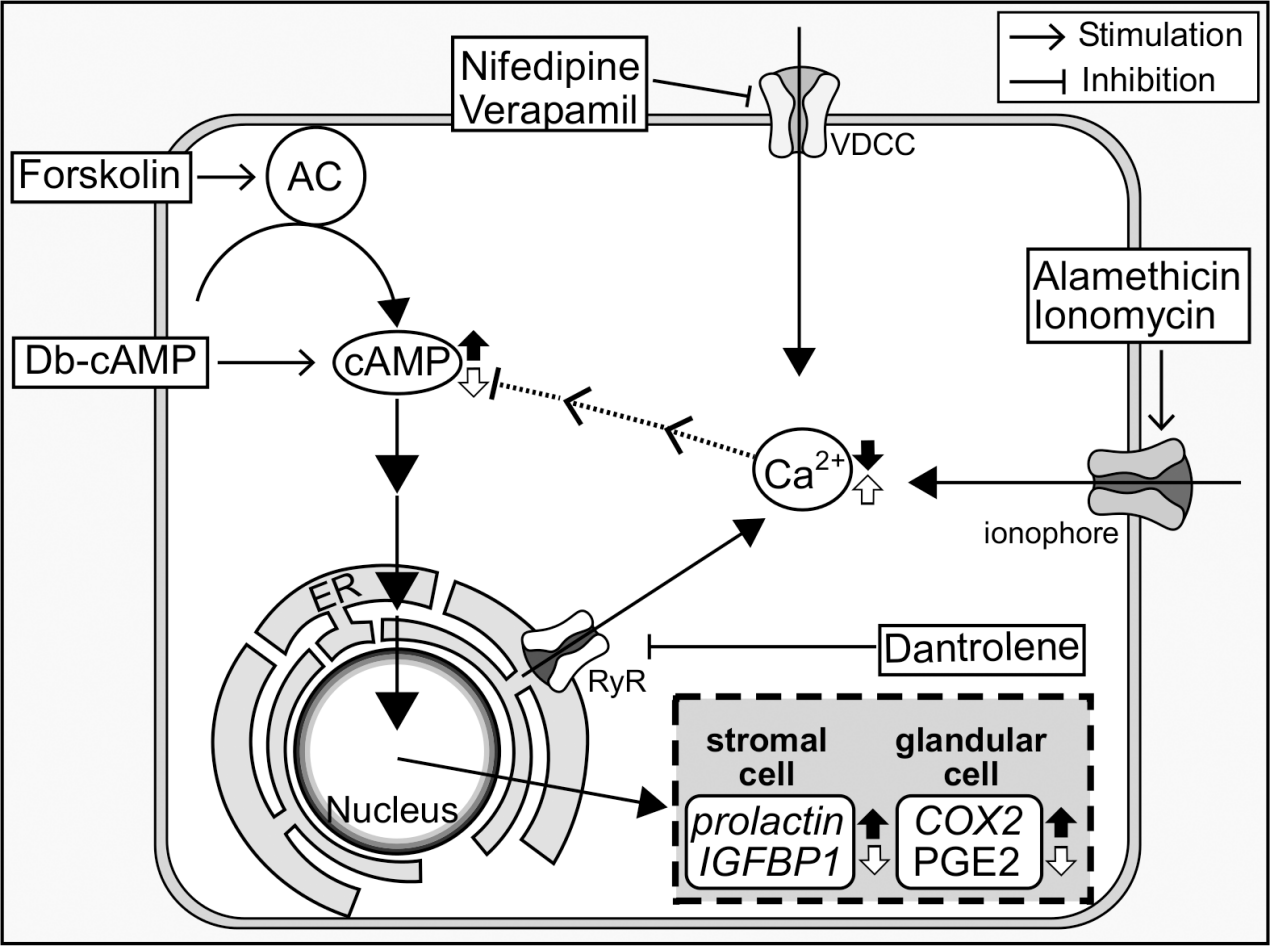
Diagram illustrating the potential role of intracellular Ca^2+^ in endometrial cells. Nifedipine and verapamil inhibit VDCC, whereas dantrolene blocks Ca^2+^ release from ER store which causes decreases in intracellular Ca^2+^ levels. Alamethicin and ionomycin promote Ca^2+^ influx through the cell membrane. Blocking Ca^2+^ causes the alternation of cAMP signaling, resulting in the down-regulation of *prolactin* and *IGFBP1*, and *COX2* and PGE2 in human endometrial stromal cell and glandular epithelial cell, respectively. Solid arrow: the effect of nifedipine, verapamil, or dantrolene. Open arrow: the effect of alamethicin or ionomycin.

In the present study, we demonstrated that forskolin or db-cAMP-stimulated decidual gene expression in human endometrial stromal cells were down-regulated by increases in Ca^2+^ levels in the cytoplasm, but up-regulated by decreases in Ca^2+^ levels. There are two calcium-mobilizing mechanisms that control Ca^2+^ concentrations in the cells, namely, Ca^2+^ influx from the extracellular regions and Ca^2+^ release from internal stores such as ER. Two Ca^2+^ ionophores, alamethicin and ionomycin, which force external Ca^2+^ influx into the cell, reduced the expression of decidual markers *prolactin* and *IGFBP1*, whereas nifedipine, verapamil or dantrolene, which decrease intracellular Ca^2+^ levels, stimulated decidual marker expression. Ca^2+^ chelater up-regulated Ca^2+^ ionophore-induced inhibition of decidual marker. In addition, continuous increases in Ca^2+^ influx decreased the cAMP concentrations in endometrial cells. These results indicated that the elevation in intracellular Ca^2+^ concentrations could have repressed *prolactin* and *IGFBP1* expression through the suppression of cAMP signaling in ESCs. Similarly, expression of implantation-related factor *COX2* and the production of PGE2 were inhibited by Ca^2+^ influx stimulators, but promoted by VDCC and RyR inhibitors in glandular epithelial EM-1 cells. In addition, Ca^2+^ chelater abolished Ca^2+^ ionophore-triggered inhibition of *prolactin* and *COX2* expression, and also enhanced forskolin-stimulated *prolactin* and *COX2* expression, suggesting that elevation of Ca^2+^ in the cytoplasm may affect negatively forskolin-stimulated *prolactin* and *COX2* expression. Fig 7 depicts the summary of our results and a possible signal pathway for changes in differentiation marker expression in endometrial stromal and glandular epithelial cells. Our findings indicated that Ca^2+^ channel blocker-triggered reduction in intracellular Ca^2+^ levels potentiated the cAMP signaling, and suggest that changes in intracellular Ca^2+^ concentrations play a role on cAMP signaling and its down-stream gene expression in decidualization and glandular maturation in the endometrium.

It was previously shown that the treatment with Ca^2+^ ionophore A23187 attenuates prolactin release in decidual cells prepared from patients in early pregnancy [25], and that Ca^2+^ channel blockers inhibit prolactin secretion in human decidual cells [26]. Ruan *et al*. [21] showed that Ca^2+^ influx increases the phosphorylation level of CREB and facilitates COX2-dependent up-regulation of PGE2. However, there were no significant changes in the levels of phosphorylated CREB protein among Ca^2+^ modulator-treated cells in this study. This probably indicates that up-regulation of decidual marker and COX-2 induced by continuous reduction in intracellular Ca^2+^ levels is not dependent on CREB activation (data not shown). Although the biochemical mechanisms associated with these differences are not known, it is possible that a progressive increase in Ca^2+^ levels through the use of pharmacological agents may possibly accelerate the degradation of cAMP, or that Ca^2+^ reduction may have led to the elevation of cAMP in undifferentiated or immature endometrial cells in this study. It has been shown that angiotensin II results in the activation of calcineurin/nuclear factor of activated T-cells (NFAT) and the elevation of COX2 expression via the elevation of intracellular Ca^2+^ in ESCs [27, 28]. In addition, prokineticin 1 (PROK1), a multifunctional secreted protein, stimulates both prolactin and IGFBP1 expression through the Ca^2+^/calcineurin/NFAT pathway in decidual cells [29–31]. Accordingly, the elevation of decidual markers induced by Ca^2+^ may be mediated partially through the pathway including calcineurin/NFAT. However, further investigation is required as to whether the reduction in cAMP levels induced by intracellular Ca^2+^ influx would influence the calcineurin/NFAT pathway. Interestingly, Ca^2+^ chelater suppressed Ca^2+^ influx inhibitor-induced up-regulation of *prolactin* and *COX2* expression in endometrial cells. These results indicate that optimal and pulsatile Ca^2+^ concentration in the cytoplasm is essential for induction of these expression. Because the spatial and temporal fluctuation of Ca^2+^ levels in the cytoplasm is necessary for diverse cellular responses including proliferation, differentiation, and gene expression, pulsatile Ca^2+^ changes in endometrial cells is probably crucial for implantation. Importantly, the conditioned media from developmentally competent embryo, but not impaired embryo induces actively intracellular Ca^2+^ oscillation [32]. Although we reported here that Ca^2+^ which may link to cAMP levels modulates differentiation of ESCs and EM-1, our study showed only pharmacological effects of Ca^2+^ modulators on decidualization and glandular maturation. It should be noted that in addition to pharmacological changes in Ca^2+^/cAMP signaling, this study was not designed to determine a role of endogenous Ca^2+^ on cAMP- or implantation-related gene expression. The mechanism that modulates intracellular Ca^2+^ oscillation would be needed to clarify.

cAMP is hydrolyzed into AMP by 3’, 5’- cyclic nucleotide phosphodiesterases (PDE) [33]. The superfamily of PDE is classified into eleven families, of which the PDE1 family is the only member to be directly activated by calcium/calmodulin (CaM), and is composed of three members, PDE1A, PDE1B, and PDE1C [34]. In human astrocytoma cells, the cAMP level was diminished by Ca^2+^ and the inhibitory effect was abolished by a selective PDE1 inhibitor [35]. The elevation of Ca^2+^ concentrations has been shown to activate PDE1A or PDE1C and to degrade cAMP in human embryonic kidney cells [36]. These findings support the possibility that Ca^2+^/CaM possibly activated PDE and then resulted in degradation of cAMP in the ESCs and EM1 cells.

In turn, five of nine AC families are directly regulated by Ca^2+^; it activates the function of AC1, AC3 and AC8 but inhibits AC5 and AC6 [33]. Ca^2+^-mediated inhibition of AC5 and AC6 reduces the cAMP concentrations in vascular smooth muscle cells [37]. AC5 and AC6 are up-regulated in pregnant human myometrium [38] and Ca^2+^ influx through VDCC attenuates skeletal muscle contraction via the inhibition of AC5 and AC6 activity [39]. However, no evidence has so far been provided as to which AC subtypes are predominantly expressed in endometrial stromal and epithelial cells. The expression of *prolactin* and *IGFBP1* was enhanced only in the presence of forskolin or db-cAMP when cells were treated with nifedipine, verapamil or dantrolene; however, treatment with each reagent without forskolin or db-cAMP had no effect on *prolactin* and *IGFBP1* expression (data not shown). The stimulatory effect of nifedipine, verapamil or dantrolene alone in EM1 cells was also similar to that in ESCs. Thus, significant effect was seen even in the condition with elevated cAMP levels. These results suggest that Ca^2+^ influx-triggered cAMP reduction in the cytoplasm could be mediated by activation of PDE1, but not by down-regulation of AC5 or AC6 in human endometrial cells. Intracellular Ca^2+^ might activate PDE1 in human endometrial stromal or glandular epithelial cells.

It has been proposed that Wnt/β-catenin signaling plays an important role in blastocyst implantation, endometrial decidualization and placental formation [40, 41]. Estrogen stimulates Wnt/β-catenin signaling during the proliferative phase of the menstrual cycle, while progesterone regulates antagonistically estrogen-induced proliferation through the inhibition of Wnt/β-catenin signaling in cell differentiation [42]. Based upon previous studies, Wnt/Ca^2+^ signaling pathway could be a crucial mediator in endometrial development [43]. It is possible that cooperative regulation of the Wnt/Ca^2+^ signaling with Wnt/β-catenin signaling could be required for the induction of proper decidualization and restricted invasion of trophoblast into the pregnant endometrium.

The exhaustion of Ca^2+^ in ER storage induces a Ca^2+^ influx, called as Ca^2+^-release activated Ca^2+^ entry (SOCE) through Ca^2+^-release activated Ca^2+^ channel (CRAC) in plasma membrane. The key molecule for the operation of SOCE has been identified as stromal interaction molecules 1 (STIM1), an ER calcium sensor. STIM2 has also been reported to be an inhibitor of STIM2-mediated SOCE [44]. Thus, regulatory mechanisms of intracellular Ca^2+^ levels are complicated and Ca^2+^ signaling cascade must be tightly controlled. Although any diseases with increased cytoplasmic calcium and the relationship between abnormal intracellular calcium and implantation failure are not known, the functional loss of intracellular molecules which regulate Ca^2+^ homeostasis such as STIM as well as IP3-sensitive Ca^2+^ channel, VDCC would affect normal Ca^2+^ oscillation in endometrial cells, as described above [32]. It is possible that endometrial cell dysfunction and implantation failure could result from defects in Ca^2+^ homeostasis and proper signaling. More research into the identification of physiological regulators for the Ca^2+^/cAMP signaling in endometrial cells will be needed to clarify the role of the Ca^2+^/cAMP pathway in uterine physiology towards pregnancy. We assume that more detail understanding of the exact regulation and function of Ca^2+^-related molecular machinery can define new clinical targets and effective therapeutic strategies.

In conclusion, we found that continuous elevation of Ca^2+^ levels lowers intracellular cAMP concentrations and the expression of implantation-related factors, and conversely that a decrease in intracellular Ca^2+^ levels promotes the expression of implantation-related factors in human endometrial cells.

## Supporting Information

S1 Fig Effects of Ca^2+^ modulators on cell viability in ESCs. ESCs plated at a density of 6 x 10^3^ cells/cm^2^ in 96 well dish were treated for 1 h with nifedipine (20 μM), verapamil (20 μM), dantrolene (20 μM), alamethicin (1 μM), or ionomycin (3, 10 μM) and then stimulated for 48 h with forskolin (15 μM). After treatment, cell viability was assessed using the WST-8 cell survival assay. After incubation with WST-8 reagent in CO_2_ incubator for 1 h, 50 μl from each well were then transferred to a 96-well microplate and read at 450 nm. The data from three independent experiments are presented. **p<0.01 *vs*. Control- no forskolin, ^##^p<0.01 *vs*. forskolin alone. Values represent the mean ± SEM.
(TIF)

S2 Fig Effects of Ca^2+^ modulators on IGFBP1 secretion in ESCs. ESCs cells were treated for 1 h with nifedipine (20 μM), verapamil (20 μM), dantrolene (20 μM), or alamethicin (1 μM) and then stimulated for 48 h with forskolin (15 μM). The IGFBP1 levels released into media were determined by ELISA. *p<0.05, **p<0.01 *vs*. forskolin alone. Values represent the mean ± SEM.
(TIF)
