# Supplementary figures and images for: Regulatory Action of Calcium Ion on Cyclic AMP-Enhanced Expression of Implantation-Related Factors in Human Endometrial Cells
Source: PLoS One. 2015 Jul 10;10(7):e0132017. doi: 10.1371/journal.pone.0132017 (PMC4498924; doi:10.1371/journal.pone.0132017)

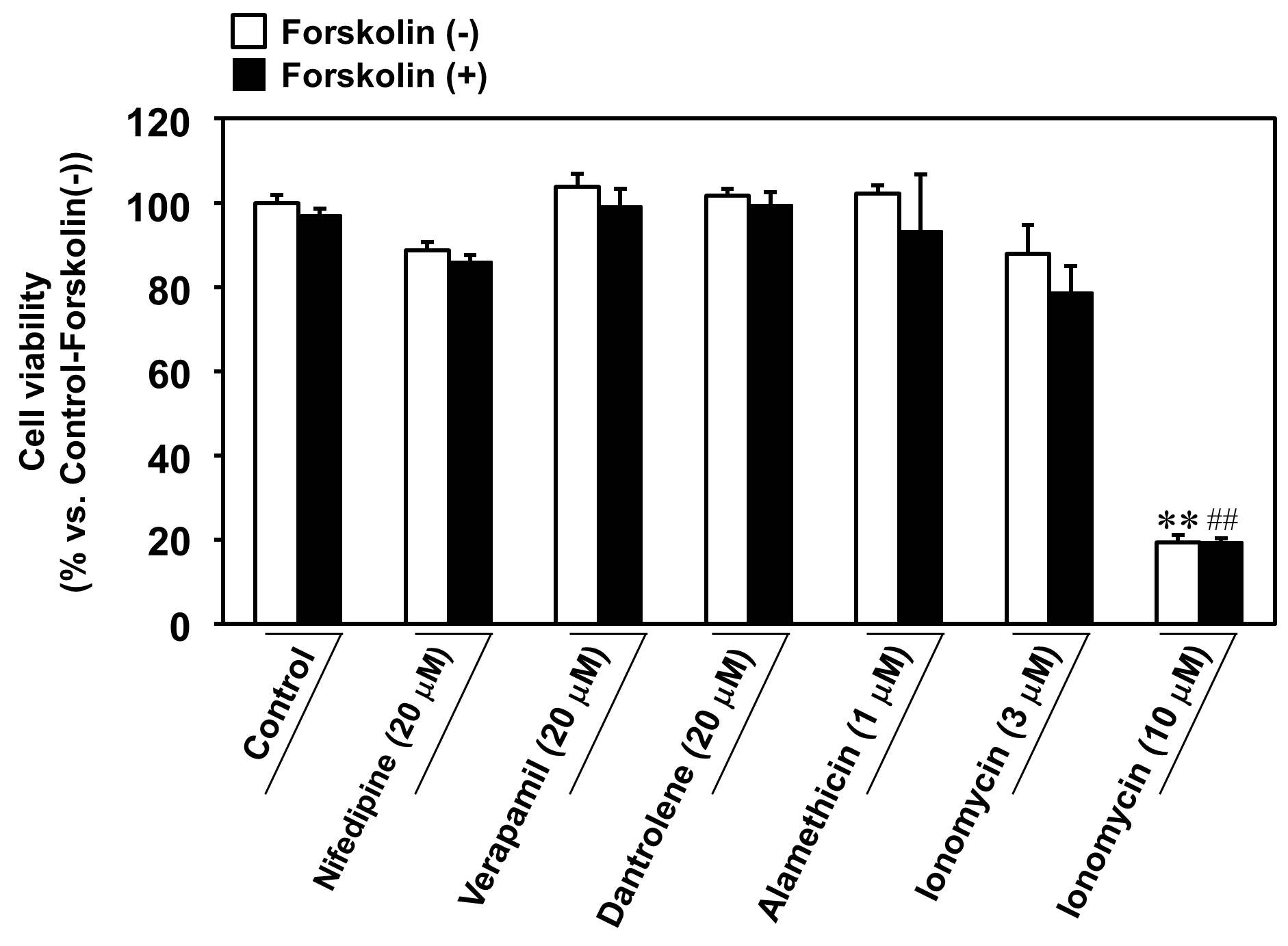

Supplement: S1 Fig — ESCs plated at a density of 6 x 10^3^ cells/cm2 in 96 well dish were treated for 1 h with nifedipine (20 μM), verapamil (20 μM), dantrolene (20 μM), alamethicin (1 μM), or ionomycin (3, 10 μM) and then stimulated for 48 h with forskolin (15 μM). After treatment, cell viability was assessed using the WST-8 cell survival assay. After incubation with WST-8 reagent in CO2 incubator for 1 h, 50 μl from each well were then transferred to a 96-well microplate and read at 450 nm. The data from three independent experiments are presented. **p<0.01 vs. Control- no forskolin, ##p<0.01 vs. forskolin alone. Values represent the mean ± SEM. (TIF) [file pone.0132017.s001.tif]

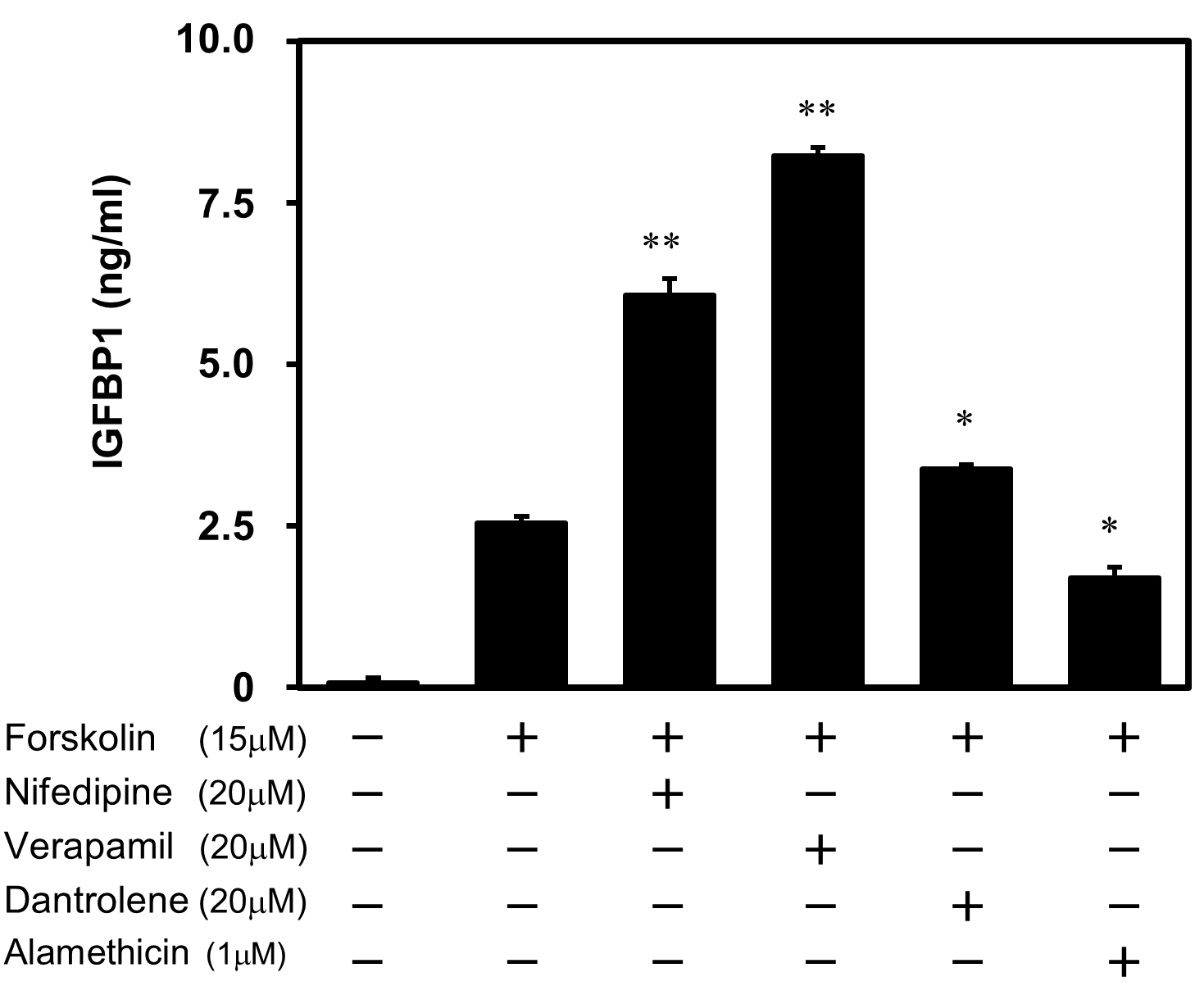

Supplement: S2 Fig — ESCs cells were treated for 1 h with nifedipine (20 μM), verapamil (20 μM), dantrolene (20 μM), or alamethicin (1 μM) and then stimulated for 48 h with forskolin (15 μM). The IGFBP1 levels released into media were determined by ELISA. *p<0.05, **p<0.01 vs. forskolin alone. Values represent the mean ± SEM. (TIF) [file pone.0132017.s002.tif]
